# Maize grain yield and grain zinc concentration response to zinc fertilization: A meta-analysis

**DOI:** 10.1016/j.heliyon.2023.e16040

**Published:** 2023-05-05

**Authors:** Dominic Mutambu, Job Kihara, Monicah Mucheru-Muna, Peter Bolo, Michael Kinyua

**Affiliations:** a Department of Environmental Sciences and Education, Kenyatta University, P.O Box 43844-00100, Nairobi, Kenya; b Alliance of Bioversity International and the International Center for Tropical Agriculture (CIAT) c/o International Centre of Insect Physiology and Ecology (ICIPE), Duduville Campus Off Kasarani Road, P.O Box 823 - 00621, Nairobi, Kenya

**Keywords:** Maize, Yield, Grain zinc, Response and agronomic bio-fortification

## Abstract

Zinc deficiency in agricultural soils is a current global agroecosystems challenge. Maize exhibits elevated susceptibility to Zn deficiency and low response to zinc fertilization. As a result, there are contradicting literature reports on the crop response to zinc fertilization. This meta-analysis synthesized the current evidence on maize response to zinc fertilization from different studies and highlighted the potential innovations to improve the crop response to zinc application. Systematic literature searches were conducted on the Web of Science and Google Scholar for peer-reviewed publications. From the selected publications, data extracted were maize grain yield and maize grain zinc concentration. The meta-analysis was conducted in R statistical environment using the *metafor* package. The ratio of means was the chosen effect size measure used. The assessment of effect size heterogeneity showed that the study effect sizes were significantly heterogeneous and also publication bias was evident. The analysis showed 17% and 25% maize grain yield and grain zinc concentration response to zinc fertilization. As a result, zinc fertilization was associated with yield increments of up to 1 t ha^−1^ and 7.19 mg kg^−1^ grain zinc concentration over the control (no zinc application). Despite the observed maize grain response to zinc application, the median concentration of grain Zn was below the 38 mg kg^−1^ recommended maize grain zinc concentration to combat human zinc deficiency (*hidden hunger*). As a result, potential innovations likely to achieve sufficient maize grain zinc content were highlighted including the use of nano-particulate zinc oxide, foliar zinc application, timing of zinc application, precision fertilization and zinc micro-dosing. Due to scanty literature on the progress of these innovations in maize, follow-up studies are recommended to evaluate their potential success in the agronomic bio-fortification of maize with zinc.

## 1 Introduction

Crop nutrients are vital for plant growth and development. Unlike primary and secondary nutrients, crop plants require micro-nutrients in small quantities. Soil deficits of the primary nutrients have widely been studied in the past largely eliminated in modern crop production systems though use of inorganic and organic fertilizers [1]. However, despite the wide adoption of the fertilizers N, P and K, in some geographies, the use of micronutrients is completely non-existent [2]. Interestingly, among the micronutrients, zinc (Zn) is the most lacking in agricultural soils and edible crop parts [3].

**Table 2:** Equations used for calculation of the pooled standard deviation and response ratios.

Formula		Reference
$\mathrm{Ln}\;\left(RoM\right)=\frac{{\bar{X}}_{Trt}}{{\bar{X}}_{Cnt}}$	*Equation 1*	[4]
$R\;\left(\%\right)=\left(e^{\mathrm{Ln}\left(RoM\right)}*100\right)-100$	*Equation 2*	[4]
$SD=SE*\;\sqrt{n}$	Equation 3	[5]
$SE=\frac{LSD}{t_{\left(0.975,n\right)}*\surd 2bn}$	Equation 4	[6]

${\bar{X}}_{Trt}$ = mean of the treatment group, ${\bar{X}}_{Cnt}$ = mean of the control group, n = sample size, b = number of replications.

Since the discovery of Zn deficiency in humans in 1961 [7], Zn deficiency has been reported to compromise the human immune system and impair cognitive growth [8]. Soil Zn deficiency, like other soil nutrients, could be attributed to numerous years of crop nutrient extraction coupled with inadequate organic or inorganic nutrient restoration [9] and the low solubility and immobilization of Zn and Fe in the soil solution [10]. Zinc deficiency is the most rampant micronutrient deficiency in agricultural soils among the soil micronutrients [11,12]. This deficiency affects the crop grain yield and Zn nutrient quality of the Zn deficiency-sensitive crops such as maize [13]. Additionally, the crop grain Zn especially in cereals is usually coupled with phytate, an anti-nutrient, making Zn from the consumed crop unavailable for uptake in the human digestive tract [14].

The genetic concentration of Zn in maize has been estimated to be 14.7–24.0 mg kg^−1^ [15]. However, the concentration required to meet human nutrition has been capped at 38 mg kg^−1^ [16]. Unfortunately, there is uncertainty of achieving both high yields and high grain Zn content in maize due to dilution effect [15]; whereby grain yield is negatively correlated with grain Zn concentration [17]. The consumption of cereals (e.g., maize) with inadequate Zn has partly been associated with high prevalence of hidden hunger (*micronutrient deficiency*) across the world [18], affecting more than 3 billion, leading to in increased morbidity and mortality, irreversible impairment to the physical and cognitive development of children, and to substantial losses in individual and national productivity [19]. The current approaches to alleviate Zn deficiency in humans include industrial food fortification, pharmaceutical supplementation, diet diversification, genetic and agronomic bio-fortification. Food fortification and supplementation are expensive and unavailable to the resource constraint populations [20], while genetic bio-fortification is a slow process, costly and coupled with uncertainties on its effectiveness in the long run [21]. Although genetic bio-fortification has been described as a superior and reliable micronutrient bio-fortification strategy [22], it cannot be a standalone approach because even the micronutrient efficient cultivars require nutrient applications to counteract crop nutrient mining from the soil [23,24]. Therefore, agronomic bio-fortification is a rapid complementary strategy to breeding strategies in alleviating hidden hunger among the resource constraint populations [21].

Fertilizer use efficiency is determined by many factors including soil characteristics [3]. Due to these inherent characteristics, soil response to nutrient application has been clustered into responsive and non-responsive soils [25]. Past research is promoting agronomic bio-fortification of Zn in staple crops by the use of zinc fertilizers (e.g. zinc sulfate, zinc sulfate monohydrate, zinc chelate, zinc oxide, zinc carbonate and zinc chloride) [26]. In some cases, however, the applied Zn is converted into crop-unavailable forms e.g. zinc carbonates in calcite soils and zinc phosphate in P-rich soils [27].

Globally, maize is among the most cultivated and preferred cereal crop for human food, animal feed or industrial use depending on the location [28]. Maize kernels contain starch, proteins, fats and essential minerals, supplying an energy density of up 365 Kcal/100 g [29]. Maize is an essential food crop in sub-Sahara Africa, Southern Asia and Latin America [29]. Maize kernel zinc levels average 20 μg/g (20 mg kg^−1^), 30% of which resides in the kernel endosperm [30]. However, maize production systems in these regions are diverse (improved germplasm, fertilizer use and other management practices) leading to variation in maize yield and profitability [31]. According to the Food and Agriculture Organization (FAO) STATS, maize yield in sub-Sahara Africa, Southern Asia and Latin America are 2.1, 3.2 and 3.0 t ha^−1^, respectively, compared the global average 5.6 t ha^−1^.

The lower maize grain yields in these regions than the rest of the world can be partly attributed to unbalanced fertilizer use [32], because of the omission of micronutrients (e.g. Zn) [[33], [34], [35]]. In maize production systems, Zn is the fourth most yield-limiting crop nutrient after N, P and K [36]. Since it is a constituent element of chlorophyll and its deficiency impairs photosynthesis thereby lowering crop yields [11]. Zn is also vital in DNA replication and gene expression in plants, synthesis of growth regulators (e.g., indole-acetic acid), pollen formation, carbohydrate metabolism and maintaining the integrity plant bio-membrane [37]. Further, according to Ref. [38] Zn is a critical enzyme co-factor and the only metal element present in all the six categories of plant enzymes.

Maize is very sensitive to soil Zn deficiency [13]. Due to its sensitivity to Zn stress [39], argues that maize is an indicator soil Zn in an area. The critical soil extractable Zn in maize production are 1.5 ppm by DTPA method, 0.8–1.17 ppm EDTA and 1 ppm by hydrogen chloride (HCl) extraction method [40]. And, the sufficient plant tissue Zn content in maize at the early growth stage should range from 20 to 70 mg kg^−1^ [41]. Based on soil DTPA extractable Zn, the probability of maize yield response to Zn is classified into three categories: high Zn response (<0.9 mg kg^−1^), medium Zn response (0.9–1.3 mg kg^−1^) and low Zn response (>1.3 mg kg^−1^) [42].

Maize response to macronutrients has been widely researched on in the past, (e.g. Refs. [33,43]). Similarly, the research on the response of maize to Zn fertilization is gaining momentum but, literature reports show inconsistent findings. Additionally, there is no recent quantitative synthesis of the response of maize to Zn application to establish the strength and direction of current evidence on maize response to Zn application. In this regard, this meta-analysis was conceptualized (i) to explore maize grain yield and grain Zn response to Zn fertilization, and to highlight the potential innovations to improve maize yield and grain content response to Zn fertilization.

## 2 Methodology

### 2.1 Literature search

Systematic literature searches were conducted in the Web of Science and Google Scholar using the search strings *“TI* = {((“Maize” OR “corn” AND “Micronutrient” OR Zinc appli*) AND (“maize grain zinc” or “content” or “concentration” or “composition")}” and “(“soil fertili*" OR “manure” OR “fertilizer”) AND (“maize grain” OR “corn grain”) AND (“nutri*" OR “zinc” OR “iron”) AND (“concentration” OR “content” OR “uptake” OR “compo*") -blood -digestibility -biofuel -greenhouse -pot –enzyme”, respectively. The inclusion/exclusion criteria for relevant publications were: (i) the study had to have been carried out under field conditions, (ii) the experiment had at least a control (no Zn applied) and treatment group (Zn applied), iii) the study must have reported grain yield and grain Zn concentration or grain yield and grain Zn uptake, and iv) The presentation of the results had to be clear for one to single out the control and Zn treatment.

**Fig. 1:**
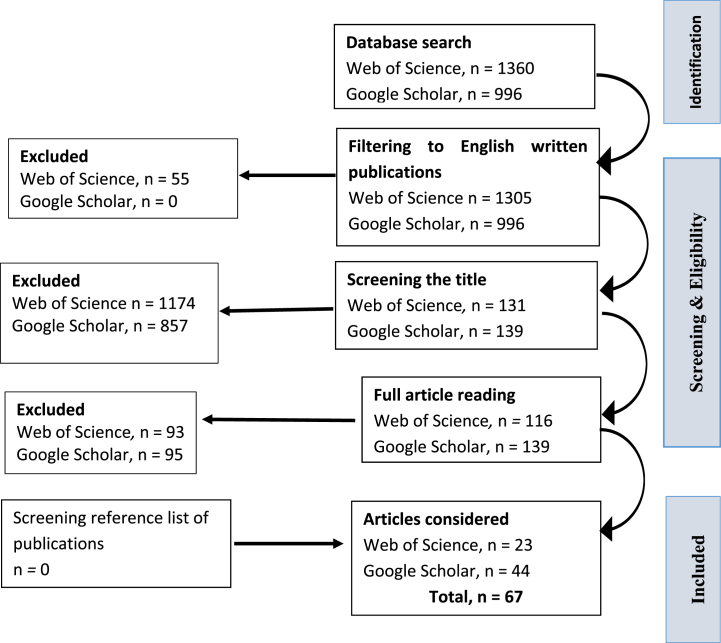
PRISMA flowchart showing the systematic literature search and publication selection criteria.

The Web of Science and Google Scholar search returned 1360 and 996 publications, respectively, published between January 2000 and 2023 (Fig. 1). The search was conducted in English . The papers were carefully read, and the decision on inclusion or exclusion was reached based on the set criteria. Whenever a conflict arose about the inclusion of a publication, a decision was reached by consensus among authors. The bibliography of the selected papers was screened to identify any additional papers. In the end, 67 publications were found fit for inclusion in this review.

### 2.2 Data extraction and exploration

A Ms excel spreadsheet was used for recording data extracted from the publications. From each study, bio-physical characteristics, variables (grain yield and grain Zn), their variance measures, and sample size were recorded. Data was extracted from the publication text, tables and published graphs and charts. In graphs where the variable data were in bars, a millimetre calibrated ruler was used to estimate the variables. The studies considered for data extraction, their geographical location, maize variety tested and treatment regime are shown in Supplementary Table 1.

### 2.3 Heterogeneity

Meta-analysis refers to a set of statistical methods for combining the effect sizes across different datasets addressing the same research question [44]. Meta-analysis allows the calculation of an overall effect size from the primary publications [45]. In the calculation of the overall effect size, meta-analyses should also assess heterogeneity, which is defined as the presence of variation in true effect sizes underlying the different studies [46].

The amount of heterogeneity (i.e. variance of the true effects, τ^2^) was estimated using the restricted maximum-likelihood estimator [47]. Between study heterogeneity (Total heterogeneity/total variability, (I^2^ statistic)) as designated by Ref. [48] was also assessed. If I^2^ = 0%, implies that the studies are homogeneous [48]. However, increasing I^2^ indicates the observed variation is due to heterogeneity not chance. In addition, Cochran's Q-statistic [49] was used to test the assumption that all evaluated studies are evaluating a similar effect size at a 90% confidence interval. According to Ref. [50] a significant Q – statistic implies that the studies don't have a common effect size, i.e. they are heterogeneous. In case any amount of heterogeneity is detected (i.e., τ^^2^>0, regardless of the results of the Q-test), a prediction interval for the true outcomes is also provided [51].

The maize grain yield meta-analysis showed that the true outcomes appeared to be heterogeneous (Q (54) = 280.99, p < 0.00, τ^^2^ = 0.01, I^2^ = 74.80%). A 95% prediction interval for the true response (%) is given by −10%–66%. Hence, although the average outcome was estimated to be positive, in some studies the true maize grain yield response to zinc application was negative. Similarly, grain Zn true outcomes were also heterogeneous (Q (62) = 605.84, p < 0.00, τ^^2^ = 02, I^2^ = 90.91%). A 95% prediction interval for the true response (%) was given by −30 to 105%. Hence, although the average outcome was estimated to be positive, in some studies the true grain Zn response to Zn fertilization was negative.

Due to the diversity of the studies involved, heterogeneity in meta-analyses is inevitable [46]. However, no amount of heterogeneity is unacceptable as long as the study's inclusion criteria used are sound and the extracted data are correct [46]. Due to the heterogeneous nature of the effect size, a random effects model was chosen for this meta-analysis [52].

### 2.4 Publication bias

In meta-analyses, funnel plot asymmetry's association with publication bias dates more than four decades ago [53]. The likely causes of publication bias include language bias - publications not written in English could be missed, citation bias – negative findings are cited less frequently [54]. Another cause of publication bias is the fact that many researchers do not like to publish negative results [55]. Therefore, publication bias may lead to an exaggerated estimate of the effect size between the studied variables [56]. However, since it is impossible to ascertain that bias affects the results of a meta-analysis, funnel plot's asymmetry should be used as sensitivity analysis rather than the predictor of the “true meta-analytic effect size” [57].

**Fig. 2:**
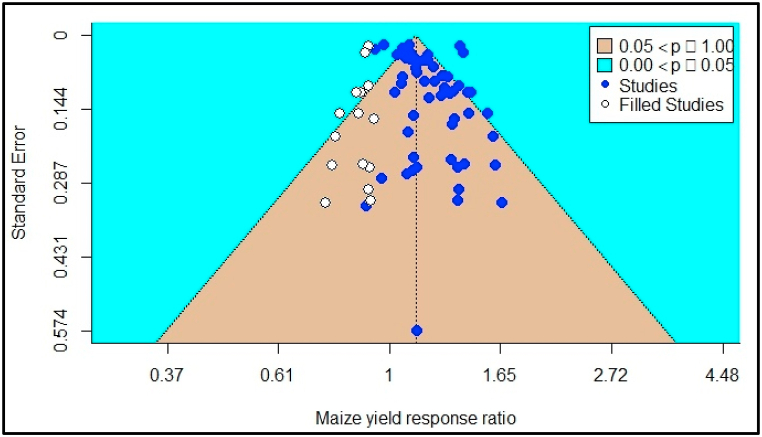
Funnel plot showing the missing publications for maize grain yield response to zinc adjusted by the trim and fill method.

**Fig. 3:**
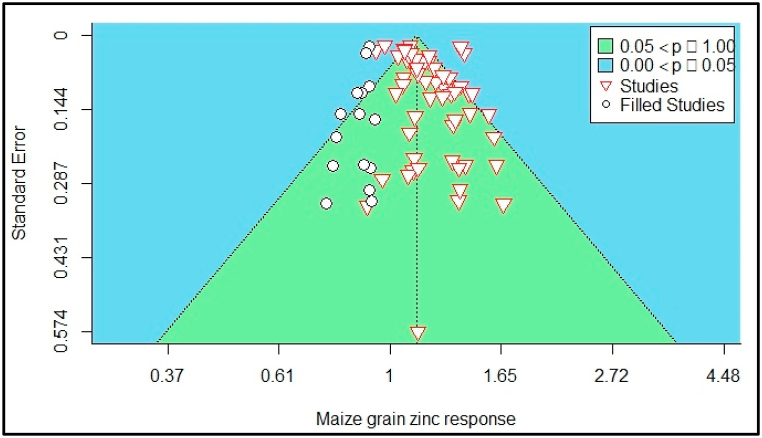
Funnel plot showing the missing publications adjusted by the trim and fill method.

Small studies and publication bias were informally evaluated by funnel plot asymmetry. Higher symmetry shows a low risk of publication bias. Then statistically confirmed by Egger's regression test [58]. The maize grain yield funnel plot of the estimates is shown in Fig. 2. Egger's regression test indicated funnel plot asymmetry (*p* = 0.01), but not rank correlation test (*Kendall's tau* = *0.10, p* = *0.30*), For the maize grain Zn, the regression test indicated funnel plot asymmetry (p = 0.00) but not the rank correlation test (Kendall's tau = 0.16, p = 0.06). Therefore, there was evidence of publication bias among the selected studies. As a result of the evidenced publication bias, the trim and fill method was used to adjust the bias as designated by Ref. [59] (Fig. 2, Fig. 3).

### 2.5 Data analysis

#### 2.5.1 Effect size measure choice and estimation

The analysis was carried out using the ratio of mean (ROM) as the effects size measure. ROM was chosen because it allows pooling of outcomes expressed in different units and comparisons of effect sizes across interventions (Zn inclusion or exclusion) [60]. The *escalc* function in *metafor* package in R Statistical Environment was used to compute the log-transformed ratio of means [61,62] (See Table 2). The natural log of the ratio of means is taken (Equation 1), which makes outcomes symmetric around 0 and yields a corresponding sampling distribution that is closer to normality [4]. For interpretation purposes, the log-transformed ROM values are converted into percentage responses by equation 2 (Table 2).

#### 2.5.2 Calculation of missing standard deviations

According to Ref. [63] the standard approach to meta-analysis of continuous outcomes requires information on the mean and either the standard deviation (SD), variance or standard error (SE) values for each treatment group. In some cases, the estimates for the measures of dispersion are not reported, instead other summary statistics such as the least significant difference (LSD) are reported [64]. To minimize bias associated with excluding those studies that do not report SDs in the meta-analysis, various ways of estimating the missing SDs have been proposed (e.g. Refs. [65,66]). In this study, missing SDs were estimated directly from the reported treatment means and sample size. Where SE was provided, SD was estimated using equation 3, in studies reporting LSDs only; the SE was estimated using equation 4 and then to SD by equation 3 whereas in studies reporting neither SE nor LSD, the SD was calculated using the STDEV function in Ms Excel with all the reported treatment means as the data array.

## 3 Results

### 3.1 Maize grain yield response to zinc

**Fig. 4:**
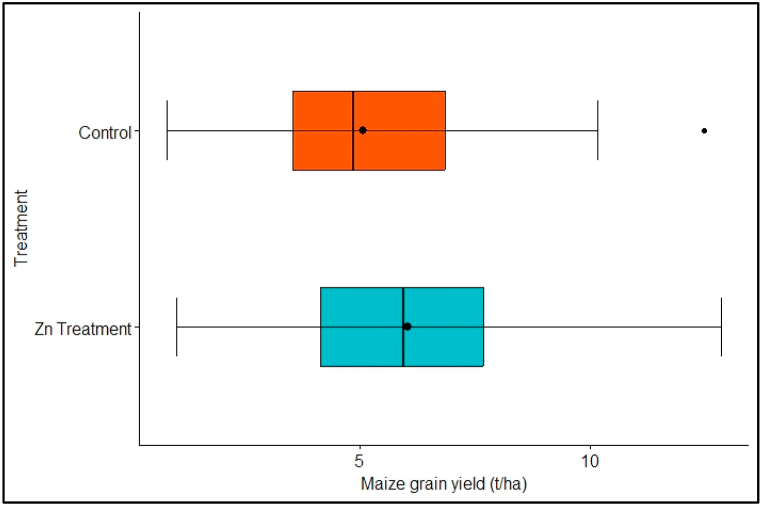
Comparison of maize grain yield in the Zn treatment and control.

Maize yield ranged from 1.02 to 12.84 t ha^−1^ and 0.83 to 12.49 t ha^−1^ in the Zn treatment and control, respectively (Fig. 4). The median maize grain yield was 6.02 and 4.93 t ha^−1^ in the Zn treatment and control, respectively (Fig. 4).

**Fig. 5:**
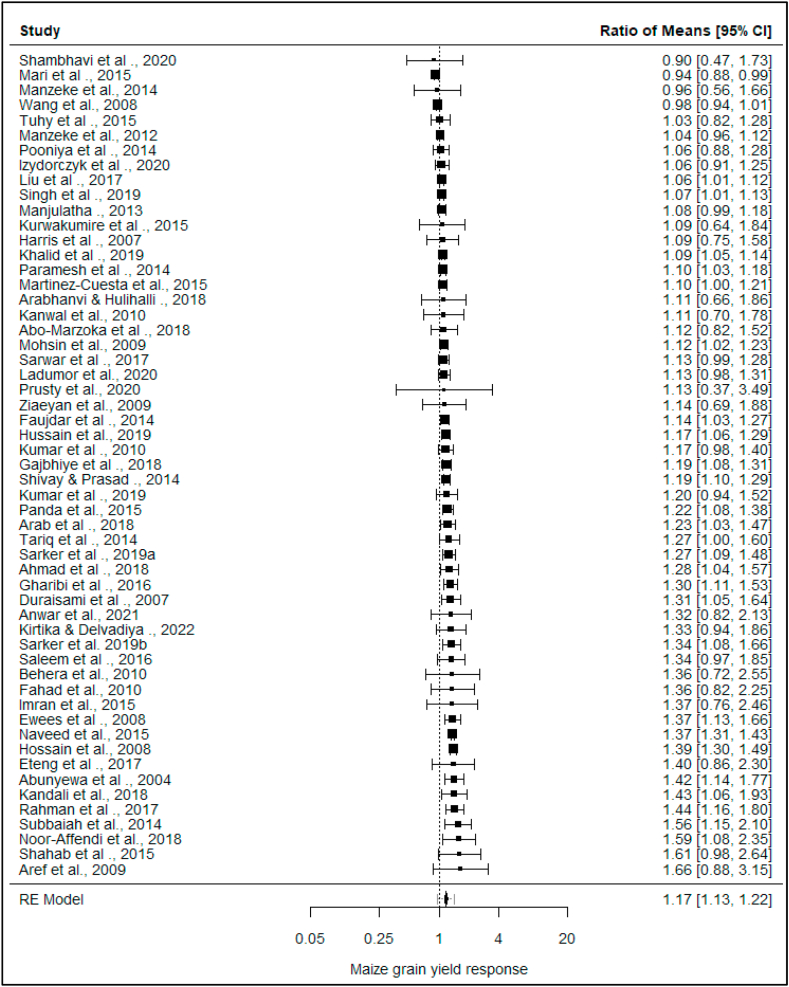
Forest plot showing the observed outcomes and the estimate of the random-effects model. The vertical dotted line shows line of no effect. Confidence interval that touches the line of no effect indicates that the study's response ratio was not statistically significant.

The observed maize grain yield response to Zn fertilization (%) ranged from −10% to 66%, with 93% of the responses being positive. The estimated average response ratio based on the random-effects model was μ^^2^ = 17% (95% CI: 13–22%). Therefore, the average outcome differed significantly from zero (*p* < *0.00*). A forest plot showing the observed maize grain yield responses and the summary response estimate based on the random-effects model is shown in Fig. 5.

### 3.2 Maize grain zinc response to micronutrient fertilization

**Fig. 6:**
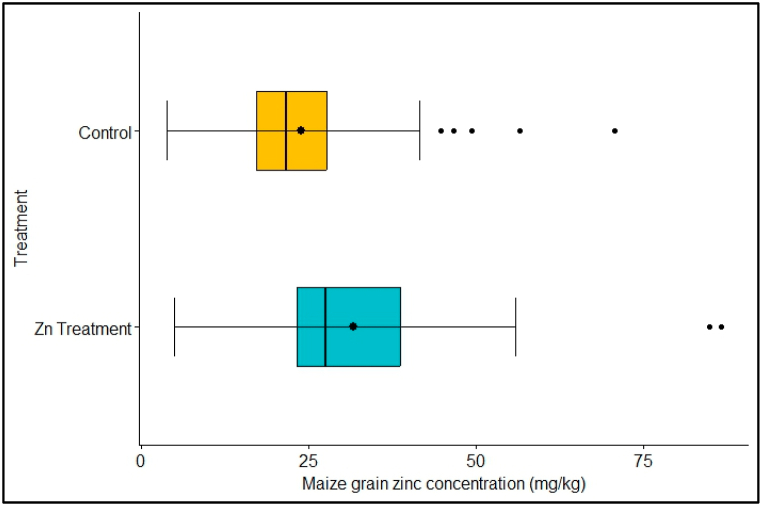
Comparison of maize grain yield in the Zn treatment and control.

The reported maize grain Zn ranged from 4.97 to 86.64 mg kg^−1^ and 3.88–70.69 mg kg^−1^ in the Zn treatment and control, respectively (Fig. 6). The median maize grain Zn concentration was 27.27 mg kg^−1^ in the Zn treatment and 21.78 mg kg^−1^ in the control.

**Fig. 7:**
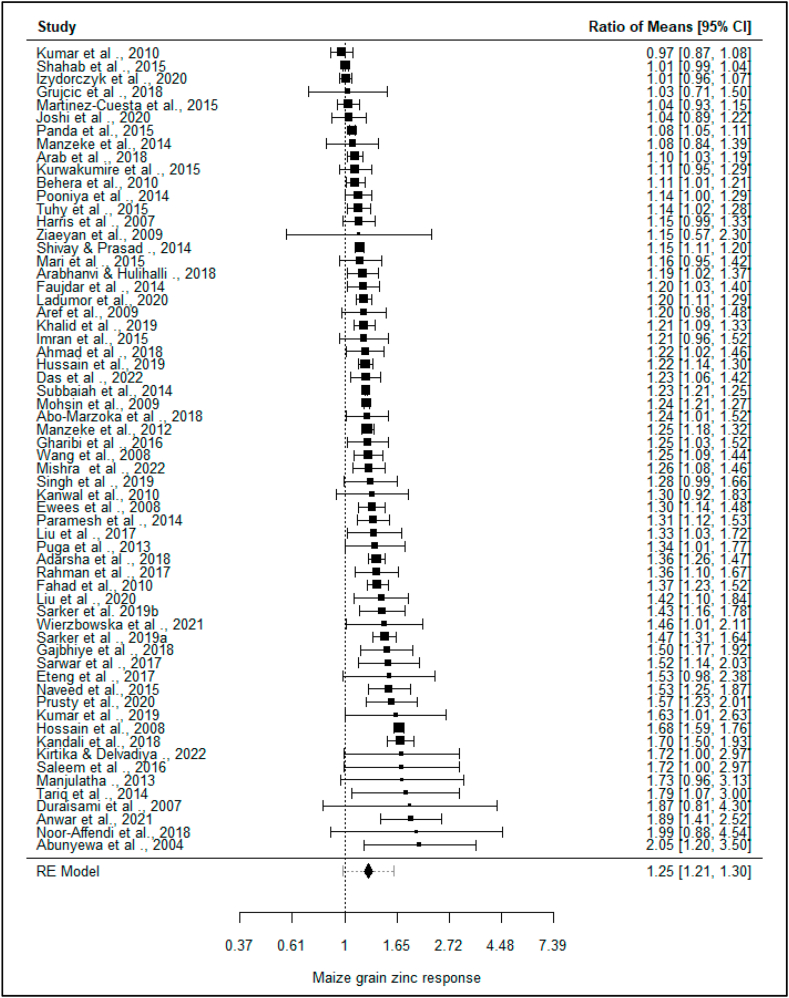
Forest plot of all the studies with their corresponding ratio of means and the respective confidence intervals. The vertical dotted line shows the line of no effect. A confidence interval that touches the line of no effect means that the study's response ratio was not statistically significant.

The estimated maize grain Zn response to Zn fertilization ranged from −30% to 105%, with only 2% of the publications reporting negative maize grain Zn response to Zn application. The estimated average response ratio based on the random-effects model was μ^^2^ = 25% (95% CI: 21.0%–30%). Therefore, the average outcome differed significantly from zero (*p* < *0.00*) (Fig. 7).

## 4 Discussion

### 4.1 Maize grain response to micronutrient fertilization

From this analysis, maize yield showed a significant response to Zn application (17%) (Fig. 5) and an associated yield increment of up to 1 t ha^−1^ (19.5%) from 5.12 to 6.12 t ha^−1^ (Fig. 4). Zinc is conventionally applied to crops together as blends with the NPK fertilizers [26]. Comparing no Zn with 15, 30 and 45 kg Zn ha^−1^ from ZnSO_4_·7H_2_O in alkaline silt loamy soil (pH 8) [67], found out that Zn significantly improved the maize grain yield than no Zn use. According to Ref. [68] the higher yields were because Zn enhanced the synthesis of carbohydrates and their transport to the site of grain production. Elsewhere [69] also opined that Zn could have positively influenced the seed set and seed weight hence greater grain yield than the no Zn application. Zn is also vital in DNA replication and gene expression in plants, synthesis of growth regulators (e.g., indole-acetic acid), pollen formation, carbohydrate metabolism and maintaining the integrity plant bio-membrane [37]. In addition, according to Ref. [38] Zn is a critical enzyme co-factor and the only metal element present in all the six categories of plant enzymes. For instance, Zn is essential for the formation and functioning of plant enzymes alcohol dehydrogenase, carbonic anhydrase and superoxide dismutase zinc-copper [70].

On the other hand, the lack of noticeable grain yield response to Zn fertilization as noted in some of the studies could be attributed to depressed Zn availability by soil chemical characteristics [37,71]. For instance, a high concentration of divalent cations in the soil increases the competition for cation exchange sites with Zn reducing crop Zn uptake [72]. Clay soils are also known to affect Zn bio-availability in the soil solution [73]. In a study on Zn solubility in varying concentrations of clay particles [74], reported that the concentration of Zn in the soil solution decreased with increasing concentration of clay particles impeding Zn availability and uptake by the studied crops.

Further, the co-application of Zn with other macro-nutrients (e.g. high rate of P could lead to P-induced Zn deficiency due to P–Zn antagonism [75]). For instance, the application of high P and low Zn reduced rice grain yield in Zn deficient field conditions [76]. The P - induced Zn deficiency results from Zn^2+^ precipitation in the presence of P to zinc phosphate [Zn_3_ (PO4)_2_] making Zn unavailable to crops for uptake in the soil and/or impeding Zn translocation from the roots to the leaves and grains [77]. Another way high P application could affect Zn uptake is by impairing AMF colonization of crop roots [78]; leading to poor Zn availability to crops [39].

### 4.2 Maize grain zinc response to Zn fertilization

The mean maize grain Zn response from this analysis was statistically significant (Fig. 7). The median grain Zn concentration was 27.27 and 21.78 mg kg^−1^ in the Zn treatment and control, respectively (Fig. 6). The average grain Zn concentration response to Zn fertilization was 25%, increasing the maize grain Zn concentration by 29.5% from 24.30 to 31.48 mg kg^−1^ (Fig. 6). As result, application of Zn to maize resulted in corresponding increment in maize grain Zn by up to 7.19 mg kg^−1^ (Fig. 6).

In the soil, crops take up Zn in the divalent ionic form (Zn^2+^) and chelated-zinc through mass flow and diffusion mechanisms by roots [79,80]. From the roots, Zn is translocated and eventually loaded to the grains. Although maize responded relatively well to Zn fertilization, majority of the studies did not report maize grain Zn content meeting the recommended 38 mg kg^−1^ for adequate human nutritional needs as designated in Ref. [16].

Maize has been shown to respond to Zn if the unfertilized control grain Zn do not exceed sufficiency threshold (18 mg kg^−1^) [81]. In this study, majority of observation in the no Zn treatment showed maize grain Zn above the sufficiency threshold. This proposition was recently re-affirmed in Argentina [82], where in five Zn deficient sites, it is reported that whenever the maize grain Zn exceeded 18 ppm in the no Zn (control) maize grain Zn did not show any noticeable response to Zn fertilization. It is also probable that the cultivars of the maize used were inefficient in absorbing the applied Zn and loading it to the grains [83] to the levels required to meet human nutritional needs.

## 5 Potential innovations to improve maize Zn response

Zinc innovations to improve Zn nutrition in staple crops are aimed at increasing use efficiencies, increasing uptake, overcoming previous zinc challenges such as the dilution effect due to increased yield, counteracting poor spread in soils and improving synergistic Zn interactions with other soil nutrients.

First, the use of zinc oxide (ZnO) nano-particles; nano-particles refers to use of engineered fertilizer granules to a diameter ranging from 0.1 to 0.5 mm and particle diameter of 2–4 μm [84,85]. Reducing fertilizer granule size may influence Zn agronomic efficiency [86] by improving fertilizer spread which has been viewed as a potential way to mitigate poor Zn spreading from larger granules [[86], [87], [88]]. Additionally, micro-granules (or rather nano-particles) have high reactivity and hence high functionality than macro-granules due to their large surface area to volume ratio [85]. Furthermore, micro-granule Zn, due to its fineness, can be applied in foliar form since the micro-granules are soluble, and also dispersible in water unlike bulky ZnO [89]. For instance, comparing the effects of varying concentrations of Zn from nano-particulate ZnO, chelated ZnSO_4_ or nano-ZnO + chelated ZnSO_4_ applied as seed treatment or foliar on pinto bean (*Phaseolus vulgaris*) seeds [86], noted that nano-particulate ZnO (0.15% Zn) significantly increased bean shoot length, internode length, shoot and root weights, yields and grain Zn content over the rest of the treatments. Additionally, micro-granulated ZnO provides both immediate and residual benefits to crop in Zn starved fields, thus lower need for continuous fertilization [85]. Although the use of nano-particulate Zn could be promising, its success in agronomic bio-fortification is dependent on crop variety, size of fertilizer particles, composition and chemical properties of the nano-materials used [90]. Additionally, there is no comparative evidence on how nano-fertilizers could potentially harm the environment and human health [90].

In circumstances where soil characteristics will impair soil-based Zn application (e.g., high bicarbonate ions), foliar fertilization can be more effective [91]. For instance, in India [92], evaluated foliar application of nano-particulate ZnO and ZnSO_4_ in non-Zn deficient field in maize and on average the yields were 15 and 42% higher in nano-particulate ZnO and ZnSO_4_ than no Zn, respectively. Additionally, a recent meta-analysis has established that foliar applied Zn in wheat increased the grain Zn by 71% compared to 25% soil application [93]. The effectiveness of foliar applied Zn would be lowered by low uptake of Zn from the plant surface and poor translocation of Zn in the plant system [94]. Other plant related factors are also associated with foliar applied utilization such as plant species and growth stage [91]. In smallholder farming systems like in SSA, uptake of foliar fertilization could be limited by its cost effectiveness [14].

Due to Zn role in plant physiology, it has been established that crop plants optimal Zn demand is at early seedling stage and at the onset of the reproductive phase [95]. Thus, establishing individual crop variety peak stages for Zn demand would inform the right fertilization time [96]. With the nutrient supplied this way, leaching has limited nutrient wastage, hence high nutrient use efficiency [97]. For instance, in wheat [98], reported that splitting Zn application showed grain Zn increase of up to 69%.

Borrowing from the operational definition of precision agriculture by Ref. [99], precision Zn fertilization entails Zn application methods, rates and fertilizer Zn species based on specific soil Zn test results and soil characteristics; especially, where other factors may significantly influence Zn availability to crops. Lastly, Zn micro-dosing. Fertilizer micro-dosing entails applying small quantities of fertilizers in the planting hole at sowing (2–6 g hill^−1^), in so doing increasing fertilizer use efficiency and yields while minimising costs [100]. Micro-dosing ensures precise and proper timing of fertilizer application [101]. However, no study was found focusing on precision Zn application and micro-dosing of Zn in maize.

The use of conventional breeding to develop cultivars with high uptake and efficient translocation of Zn to maize grain [102]. Breeding strategies can also reduce the phytate concentration in the grain thus increasing Zn bioavailability for human nutrition [103]. However, breeding a slow process, costly and coupled with uncertainties (Cakmak, 2008) especially in the current era of climatic variation and change. Additionally, it cannot be a standalone approach because even the micronutrient efficient cultivars would require nutrient applications to counteract crop nutrient mining from the soil [23,24].

## 6 Conclusion

This meta-analysis sought to investigate the expected maize grain yield and Zn density as influenced by Zn application. It is apparent that maize grain yield and grain Zn density responds to Zn application by up to 17 and 25%, respectively. However, despite the observed grain Zn response, the average grain Zn concentration in the Zn treatment was 31.48 mg kg^−1^, 6.52 mg kg^-1^ below the recommended 38 mg kg^−1^. Therefore, more research is needed to evaluate the factors that need to be addressed for agronomic bio-fortification of Zn to achieve the recommended 38 mg kg^−1^ grain Zn level in maize.

## Declaration of competing interest

The authors have no conflict of interest to declare.

## Appendix A Supplementary data

Supplementary Table 1.docx Supplementary Table 1.docx
