# Supplementary material for: Maize grain yield and grain zinc concentration response to zinc fertilization: A meta-analysis
Source: Heliyon. 2023 May 5;9(5):e16040. doi: 10.1016/j.heliyon.2023.e16040 (PMC10192774; doi:10.1016/j.heliyon.2023.e16040)
Supplement: Supplementary Table 1.docx [file mmc1.docx]

**Supplementary Table 1:** Studies included in the meta-analysis, country of the study, treatment regimes, maize variety studied, and the response variable reported

| **Study** | **Source** | **Country** | **Maize variety studied** | **Treatment**  **regime** | **Response**  **Variable** |
| --- | --- | --- | --- | --- | --- |
| Abo-Marzoka et al ., 2018 | Egyptian Journal of Agricultural Sciences, 69(1), 63-72 | Egypt | Single hybrid Sc10 | NP+Mn | 1,2 |
| Abunyewa et al ., 2004 | Asian J. Plant Sci | Ghana |  | NPK+Mn | 1,2 |
| Adarsha et al ., 2018 | Journal of Pharmacognosy and Phytochemistry | India | NK-6240 | NPK+Mn | 2 |
| Ahmad et al ., 2018 | Current Agriculture Research Journal, 6(1) | Pakistan |  | NPK+Mn | 1,2 |
| Anwar et al., 2021 | Asian Journal of Agriculture and Biology | Pakistan | Soni dharti-626 | NPK+Mn | 1,2 |
| Arab et al ., 2018 | Journal of Soil Sciences and Agricultural Engineering, 9(9) | Egypt | SC168 | Mn | 1,2 |
| Arabhanvi & Hulihalli ., 2018 | Int.J.Curr.Microbiol.App.Sci.2018.7(2) | India | Hybrid sugar 75 | NPK+Mn | 1,2 |
| Aref et al., 2009 | Journal of American Science | Iran | Single Cross 401 | NPK+Mn | 1,2 |
| Behera et al., 2010 | Indian Journal of Agricultural Sciences 79(4) | India | Ganga Safed 2 | NPK+Mn | 1,2 |
| Das et al ., 2022 | International Journal of Economic Plants 9(3) | India |  | NPK+FYM+Mn | 2 |
| Duraisami et al ., 2007 | Madras Agricultural Journal, 94(7/12) | India |  | FYM+Mn | 1,2 |
| Eteng et al ., 2017 | International Journal of Research Studies in Science, Engineering and Technology | Nigeria | Bende-W' Oba supper II-Y, Pioneer Seed-W, TZSR-Y, Oba Super I-W | NPK+Mn | 1,2 |
| Ewees et al ., 2008 | Research Journal of Agriculture and Biological Sciences, 4(5): | Egypt | single cross 10 hybrid | FYM+Mn+Inocculation | 1,2 |
| Fahad et al., 2010 | CLEAN–Soil, Air, Water, 43(10) | Pakistan | Pop 2004B, Pop 2006, Azam, Pahari, Sarhad (W) | NPK+Mn | 1,2 |
| Faujdar et al ., 2014 | Asian Journal of Soil Science | India | Pratap Makka-5 | NPK+Mn | 1,2 |
| Gajbhiye et al ., 2018 | Int. J. Curr. Microbiol. App. Sci | India |  | NPK+FYM | 1,2 |
| Gharibi et al ., 2016 | Journal of Plant Production, 7(5) | Egypt | hybrid 2031 | NP+Mn | 1,2 |
| Grujcic et al ., 2018 | ournal of Trace Elements in Medicine and Biology, 49 | Bosnia and Herzegovina |  | NPK+Mn | 2 |
| Harris et al ., 2007 | Field Crops Research | Pakistan | Kissan-90 | Mn | 1,2 |
| Hossain et al., 2008 | Plant and Soil, 306(1) | Bangladesh | Pacific 984 | Mn | 1,2 |
| Hussain et al., 2019 | Agronomy | Pakistan | Syngenta NT662 | NPK+Mn | 1,2 |
| Imran et al., 2015 | Journal of Plant Nutrition and Soil Science | Pakistan | Neelam, Monsanto DK-6142 | | 1,2 |
| Izydorczyk et al ., 2020 | Science of The Total Environment | Poland | LG 32.58 | FYM+Mn | 1,2 |
| Joshi et al ., 2020 | Indian Journal of Agricultural Sciences, 90(9) | India | PEEHM 5 | NPK+Mn | 2 |
| Kandali et al., 2018 | Journal of the Indian Society of Soil Science | India | PAC 740 | NPK+Mn | 1,2 |
| Kanwal et al., 2010 | International Journal of Agriculture & Biology | Pakistan | Golden, FHY-421 | NPK+Mn | 1,2 |
| Khalid et al ., 2019 | Semina: Ciências Agrárias | Pakistan | YSM-112, DK-6525 | NPK+Mn | 1,2 |
| Kirtika & Delvadiya ., 2022 | The Pharma Innovation Journal | Pakistan | hybrid919 | NPK + Mn | 1,2 |
| Kumar et al ., 2010 | Mysore Journal of Agricultural Sciences | India |  | Mn | 1,2 |
| Kumar et al ., 2019 | Journal of Pharmacognosy and Phytochemistry | India | Shaktiman 1, Shaktiman 2, Shaktiman 3 | FYM+Mn | 1,2 |
| Kurwakumire et al ., 2015 | Agronomy Journal | Zimbabwe | SC513 | NPK+Mn | 1,2 |
| Ladumor et al., 2020 | Maydica, 64(3) | India | GM-6 | NPK+Mn | 1,2 |
| Liu et al ., 2017 | Plant Soil (Springer) | China | Zhengdan 958 | NPK+Mn | 1,2 |
| Liu et al ., 2020 | Frontiers in Plant Science | China | Zhengdan 958 | NPK+Mn | 2 |
| Manjulatha ., 2013 | Maize Journal | India |  | NPK+Mn | 1,2 |
| Manzeke et al., 2012 | Plant and Soil | Zimbabwe | SC513 | NP+Mn+FYM, NP+Mn | 1,2 |
| Manzeke et al., 2014 | Field Crops Research | Zimbabwe |  |  | 1,2 |
| Mari et al ., 2015 | American Journal of Plant Sciences | Brazil | Dow AgroSciences 2B710 | NPK+Mn | 1,2 |
| Martinez-Cuesta et al., 2015 | Journal of Plant Nutrition | Argentina | Dk7210V3P, NK840TDTG, LT632RR, LT626VT3P | NP+Mn | 1,2 |
| Mishra et al ., 2022 | The Pharma Innovation Journal 2022; 11(4): 1669-1676 | India | Azad Uttam | NP+Mn | 2 |
| Mohsin et al., 2009 | The Journal of Animal & Plant Sciences | Pakistan | Pioneer 30-Y-87, DK-919 | NPK+Mn | 1,2 |
| Naveed et al., 2015 | European Journal of Agronomy | Pakistan | Hybrid-6585 | NPK+Mn+FYM | 1,2 |
| Noor-Affendi et al., 2018 | Communications in Soil Science and Plant Analysis | Malaysia | Mas Madu | NPK+Mn | 1,2 |
| Panda et al., 2015 | Research Journal of Agricultural Sciences | India | PKV Shatak | NPK+Mn | 1,2 |
| Paramesh et al ., 2014 | Indian Journal of Agronomy | India | PEHM 2 | NPK+Mn | 1,2 |
| Pooniya et al ., 2014 | Experimental Agriculture | India | HQPM 1 | NPK+Mn | 1,2 |
| Prusty et al., 2020 | Journal of the Indian Society of Coastal Agricultural Research | India | High Shell | NPK+Mn | 1,2 |
| Puga et al ., 2013 | Cien. Inv. Agr. 40(1):97-108. 2013 | Brazil | Impacto | NPK+Mn |  |
| Rahman et al ., 2017 | Bangladesh Journal of Botany, 46(1 Suppl.) | Bangladesh | BHM 5 | NPK+Mn | 1,2 |
| Saleem et al ., 2016 | Journal of Agriculture and Ecology Research International, 7(4) | Pakistan | Hybrid-919 | NPK+Mn | 1,2 |
| Sarker et al ., 2019a | Communications in Soil Science and Plant Analysis | India | Joi Kishan | NPK+Mn | 1,2 |
| Sarker et al. 2019b | Communications in Soil Science and Plant Analysis | Bangladesh | | NPK+Mn | 1,2 |
| Sarwar et al ., 2017 | Journal of Plant Nutrition | Pakistan | NARC-2704 | NPK+Mn | 1,2 |
| Shahab et al ., 2015 | Environment and Plant Systems | Pakistan |  | Mn | 1,2 |
| Sharma et al ., 2021a | Journal of Plant Nutrition | India |  | NPK+FYM+Mn | 2 |
| Sharma et al., 2021b | Journal of Plant Nutrition | India |  | NPK+FYM+Mn | 1 |
| Shivay & Prasad ., 2014 | Egyptian Journal of Biology | India | High Quality Protein Maize 1 | NPK+Mn | 1,2 |
| Singh et al ., 2019 | Journal of Pharmacognosy and Phytochemistry | India | PMH-1 | Mn | 1,2 |
| Stewart et al ., 2019 | Journal of Plant Nutrition | USA |  | Mn | 1,2 |
| Subbaiah et al., 2014 | Journal of Agricultural and Food Chemistry | India | DHM-117 | NPK+Mn | 1,2 |
| Tariq et al ., 2014 | American Journal of Plant Sciences | Pakistan | Monsanto 6525, Pioneer-32F 10, Hycorn 8288 | NPK+Mn | 1,2 |
| Tuhy et al ., 2015 | Open Chemistry | Poland | KOSMO 230 | NPK+Mn+Inocculation | 1,2 |
| Wang et al., 2008 | Field Crops Research | China | Zhendan 958 | Mn | 1,2 |
| Wierzbowska et al ., 2021 | Journal of Elementology, 26(4) | Poland | NK Borago | NPK+Mn | 2 |
| Ziaeyan et al., 2009 | International Journal of Plant Production | Iran |  | NP+Mn | 1,2 |

N – nitrogen, P- phosphorus, Mn - micronutrient zinc, 1 – stands for maize grain yield, 2 - stands for maize grain zinc concentration
